# Current challenges in TAVI: neo-commissural alignment to mimic more physiologic valve implantation

**DOI:** 10.20517/2574-1209.2020.55

**Published:** 2020-12-10

**Authors:** Aditya Sengupta, Sophia L. Alexis, Jason C Kovacic, Gilbert H. L. Tang

**Affiliations:** 1 Department of Cardiovascular Surgery, Icahn School of Medicine at Mount Sinai, New York, NY 10029, USA.; 2 Division of Cardiology, Department of Medicine, Icahn School of Medicine at Mount Sinai, New York, NY 10029, USA.

**Keywords:** Aortic stenosis, commissural alignment, aortic valve, coronary artery disease

## Abstract

Commissural alignment during transcatheter aortic valve implantation (TAVI) has important clinical implications as TAVI expands to younger patients in whom lifetime treatment of aortic valve disease and coronary artery disease is of particular importance. Numerous studies have shown that lack of commissural alignment may adversely affect coronary reaccess and the feasibility of redo-TAVI in this patient population. To assess the risk of commissural misalignment more accurately, we have pioneered and validated the use of a preprocedural imaging protocol that determines valve orientation using multi-detector computed tomography-fluoroscopy co-registration. Furthermore, we have shown that a modified delivery system insertion technique during initial valve deployment results in improved commissural alignment and reduced coronary artery overlap following TAVI with a self-expanding device. However, numerous unanswered questions remain about the impact of commissural misalignment on balloon-expandable valve-in-valve TAVI, especially in patients with unfavorable aortic root anatomy. It is imperative that clinicians consider these anatomic, device-related, and procedure factors, among others, when evaluating patients for transcatheter therapies.

## INTRODUCTION

With the conclusion of the recent low-risk trials, transcatheter aortic valve implantation (TAVI) has now been approved for patients with symptomatic, severe aortic stenosis across all surgical risk categories^[1,2]^. Amidst these rapidly evolving clinical indications, several issues related to the long-term durability and feasibility of TAVI in younger patients have been raised by the structural heart community. One such example is transcatheter heart valve (THV) orientation during initial deployment and its impact on commissural alignment. Whereas direct visual inspection and excision of native leaflets during surgical aortic valve replacement (SAVR) readily allows alignment of the surgical valve commissures with the native commissures, commissural alignment with THVs during TAVI is far more inconsistent and random^[3]^. Commissural malalignment may lead to varying degrees of overlap between the neo-commissural posts and coronary arteries^[4,5]^. Furthermore, experimental models have shown that THV leaflet stress and central aortic regurgitation (AR) may be exacerbated with suboptimal commissural alignment^[3,6]^. These findings have significant clinical implications for younger patients who have an increased lifetime risk of complications of aortic valve disease and coronary artery disease. Given that coronary reaccess and redo-TAVI will become more prevalent in the future, achieving commissural alignment during initial TAVI may impact the feasibility of both these procedures. Here, we review some of the salient features of neo-commissural alignment and offer our perspectives on how to achieve a more physiologic valve implantation.

## CORONARY REACCESS

Contemporary THV device and delivery system designs do not allow for consistent and precise commissural alignment. Following initial TAVI, an obstructive commissural post may significantly hinder future coronary access by extending above, in front of, or through the coronary ostia^[5]^. Coronary reaccess is impeded not just by the obstructive THV stent frame, but also by an *in situ* barrier formed by the native aortic leaflets. Thus, the anatomy relating the aortic root to the valve stent frame must be thoroughly evaluated. The coronary arteries are easily reached when the sinotubular junction (STJ) or coronary ostia are situated distal to the transcatheter valve stent frame. However, when either of these structures is located below the THV frame, the TAVI operator would need to cross the stent frame to access the coronary arteries. This may not be a major issue when using THVs with short stent frame heights provided that the native aortic valve does not obstruct the open cells of the stent frame^[7]^.

## REINTERVENTION AFTER INITIAL TAVI

Commissural misalignment during initial TAVI also jeopardizes the success of future valve-in-procedures for the treatment of prosthetic valve failure. While TAVI-in-SAVR is a relatively simple procedure, redo-TAVI in the current era is associated with a number of anatomic risks, including coronary obstruction, that are exacerbated in the absence of neo-commissural alignment^[8]^. Thus, consideration of the THV leaflet height within the anatomic boundaries of a patient’s aortic root becomes crucial when evaluating him or her for redo-TAVI. As before, if the leaflets lie proximal to the coronary ostia, valve-in-valve TAVI should be feasible. If not, an adequate margin between the valve stent frame and sinotubular junction is imperative to prevent sinus sequestration and coronary obstruction^[7]^.

In recent years, the BASILICA (Bioprosthetic or native Aortic Scallop Intentional Laceration to prevent Iatrogenic Coronary Artery obstruction) procedure has been successfully applied in TAVI-in-SAVR cases with a high risk of coronary obstruction^[9]^. However, this procedure may not be as easily performed during redo-TAVI, especially with supra-annular THVs since leaflet splitting can be impeded by the valve stent frame. In cases of commissural misalignment that appose the commissural post to a coronary ostium, the BASILICA technique may not completely eliminate the possibility of coronary obstruction in redo-TAVI.

## IMAGING-BASED ASSESSMENT OF THV ORIENTATION

Accurate imaging is essential for preprocedural planning. To assess the risk of commissural misalignment and severe coronary overlap, our group pioneered the technique of determining THV orientation using multi-detector computed tomography (MDCT)-fluoroscopy co-registration^[10]^. Briefly, we begin by measuring the en-face angle between the left main and right coronary arteries using MDCT. Next, using the 3Mensio Valves software (Pie Medical Imaging version 9.1, Maastricht, Netherlands), we capture the THV orientation in the three-cusp coplanar fluoroscopic view and co-register it onto our coplanar MDCT axial images. We can then superimpose a virtual image of either the SAPIEN 3 THV (Edwards Lifesciences LLC, Irvine, CA, USA) or the Evolut THV (Medtronic Inc., Minneapolis, MN, USA) over the axial MDCT annular and sinus of Valsalva images derived from the 3Mensio Valves software. This allows us to determine the degree of overlap between the neo-commissures and the coronary ostia^[11]^. Note that the use of various third-party software systems introduces the risk of operator bias that has to be taken into consideration when performing the aforementioned analyses.

## IMPACT OF THV TYPE AND CONTEMPORARY RESULTS

We have previously used the aforementioned co-registration technique to assess the relationship between THV deployment orientation and commissural alignment as part of the ALIGN-TAVR (Alignment of Transcatheter Aortic-Valve Neo-Commissures) study. Here, > 30%-50% of the 828 patients who underwent TAVI from 2016–2019 (483 SAPIEN 3, 245 Evolut, and 100 ACURATE-neo) had overlap with at least one coronary artery. More importantly, commissural alignment was unaffected by initial deployment orientation of the SAPIEN 3 THV, but was significantly improved by specific initial orientations of the Evolut and ACURATE THVs^[12]^. The nuances between the two main types of commercially available THVs in the context of the ALIGN-TAVR and other contemporary studies are discussed below.

### SAPIEN 3

The balloon-expandable SAPIEN 3 valve can have one commissure crimped at the 3, 6, 9, or 12 o’clock orientation relative to the delivery catheter to track the initial deployment orientation. In the ALIGN-TAVR study, commissural alignment was not improved by crimping the SAPIEN 3 THV at each of the aforementioned orientations^[12]^. We speculated that this may be due to the flexibility of the delivery catheter as it courses through the aorta. Fortunately, the unassuming profile of SAPIEN 3 stent frame renders commissural alignment less pertinent for coronary reaccess as wires and catheters can engage the coronary ostia above and through the top row of the stent frame. Coronary access can nevertheless be challenging in certain cases where the SAPIEN 3 stent frame protrudes beyond a narrow STJ^[13,14]^.

Similar findings were reported by Rogers *et al*.^[15]^ in their study of 137 low surgical risk patients from the LRT (Low Risk TAVR) trial (NCT02628899) who underwent balloon-expandable TAVI. Using post-TAVI MDCT analysis, the authors found that 9%-13% of patients displayed high-risk alignment due to the valve stent frame extending distal to the coronary ostia and an obstructive commissural post. Similar to the ALIGN-TAVR study, commissural alignment was not significantly influenced by intentional crimping of the transcatheter valve. The THV stent frame protruded beyond the STJ in 21% of patients, and the THV-STJ margin was < 2 mm in 13% of patients. Patients with THV-STJ margin < 2 mm were deemed anatomically unsuitable for redo-TAVI given the excessive perceived risk of coronary obstruction^[15]^. Despite limited generalizability, the findings from this anatomic simulation corroborate the results from our pilot angiographic study that show that redo-TAVI may not be possible in > 20% of SAPIEN 3 patients and more than half of those with unfavorable aortic root anatomy (sinus height < THV height)^[16]^.

### Evolut

**Figure 1.**
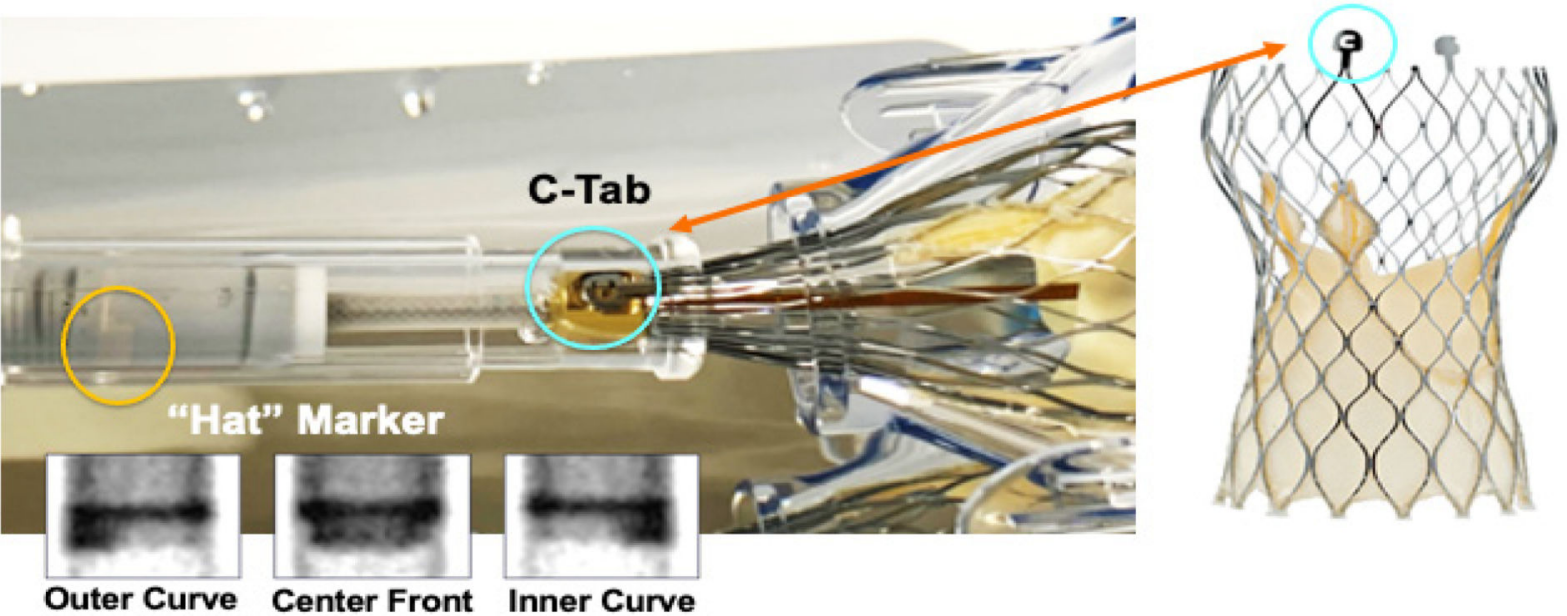
Orientation-specific deployment of the evolut transcatheter heart valve

**Figure 2.**
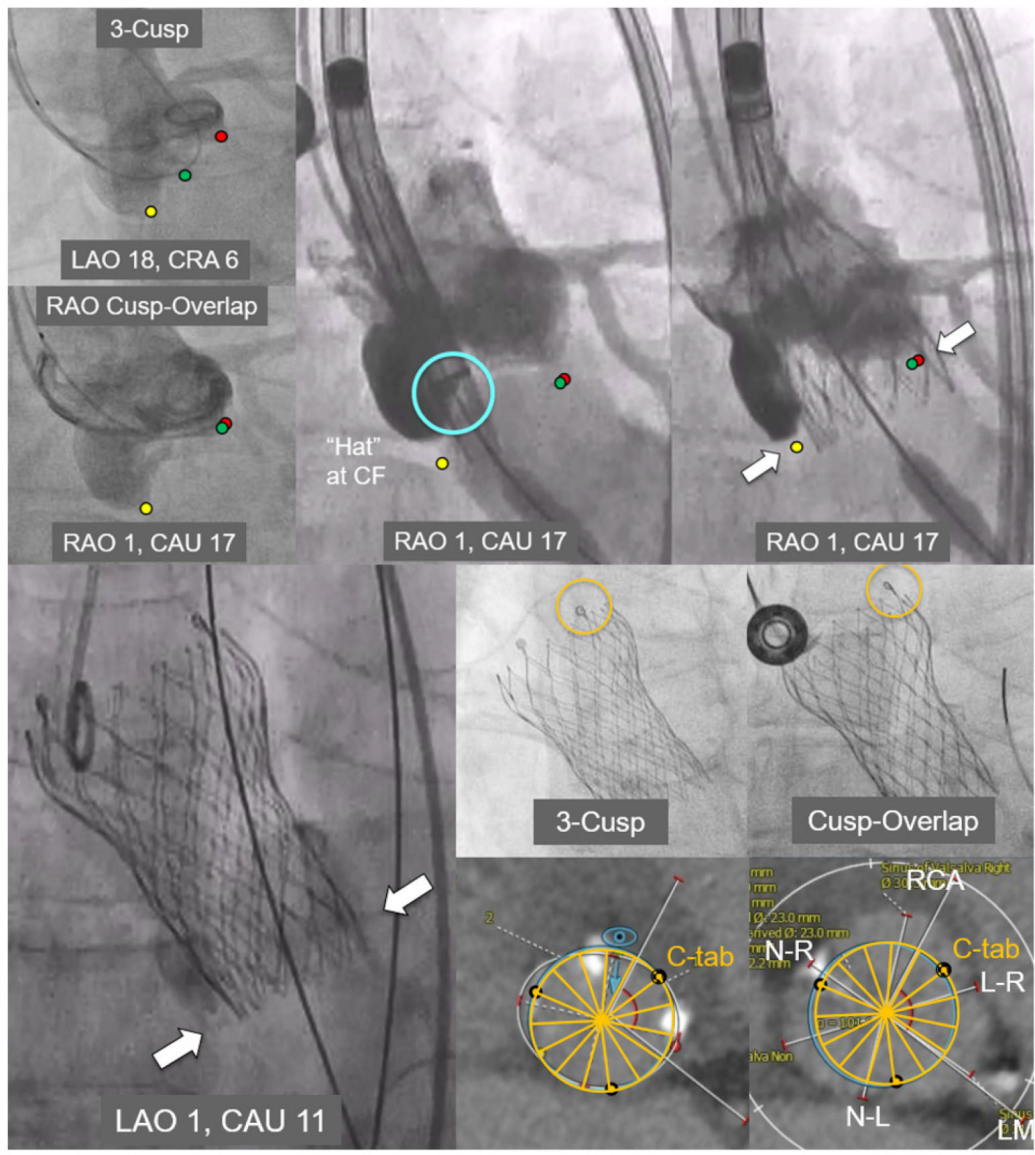
Optimization of commissural alignment with the evolut system

Commissural alignment is particularly important for facilitating coronary reaccess following Evolut TAVI since this supra-annular valve extends above the STJ and coronary ostia. The Evolut THV has a unique “Hat” marker that can be oriented during initial deployment in one of four positions: anterior or posterior in the center of the deployment device [center front (CF) or center back (CB)] or in the inner (IC) or outer curve (OC) of the aortic annulus [Figure 1]. In the ALIGN-TAVR study, positioning the Evolut “Hat” at OC or CF during valve deployment resulted in significantly less severe coronary overlap than IC/CB positioning [Figure 2]. Furthermore, tracking the “Hat” marker at the outer curve of the descending aorta improved deployment at the OC of the aortic annulus from 70.2% to 91.6% (*P* = 0.002) and reduced coronary artery overlap by 36%-60% (*P* < 0.05). Perhaps most importantly, we found that the best method to achieve OC/CF “Hat” orientation involves starting with the flush port at 3 o’clock during insertion of the delivery catheter into the femoral artery^[12]^.

The above findings were validated in a recent analysis of the impact of delivery system insertion technique on commissural alignment. Here, 154 of 249 patients from the Evolut Low Risk trial (NCT02701283) CT sub-study who underwent transfemoral TAVI using the conventional delivery system insertion technique (flush port at 12 o’clock) were compared to 240 patients from our institution who underwent deployment using a modified technique (flush port at 3 o’clock). Unsurprisingly, the modified technique significantly improved “Hat” marker orientation at OC/CF during initial deployment (93.1% *vs*. 69.6%, *P* < 0.001), improved commissural alignment, and reduced severe coronary overlap (left main artery: 15.2% *vs*. 27.7%, *P* = 0.004; right coronary artery: 11.8% *vs*. 27.7%, *P* < 0.001)^[17]^. Note that we insert the delivery catheter at 3 o’clock but let the system self-rotate as it goes inside the body. We do not force the system to maintain the 3 o’clock position as it tracks to the annulus, just like we never force the catheter to maintain the 12 o’clock position when using the conventional technique. We do not recommend force-rotating the delivery catheter inside the patient given the risk of damaging the catheter.

The mechanisms underlying the improved results with the modified insertion technique remain nebulous but may be related to the location of the spine within the Evolut delivery system. Significant columnar rotation may be limited by the two spines inside the delivery system as this is advanced along the aorta, thus maintaining the “Hat” orientation at THV deployment. This technique should only be performed in the descending aorta, and caution other operators that further validation is required to ascertain its safety^[12]^.

Similar to the Evolut THV, the self-expanding JenaValve system makes use of clipping mechanisms that enhance valve positioning and fixation. It remains to be seen whether this anatomic positioning translates into a reduced risk of coronary artery overlap^[14]^.

## CONCLUSION

Achieving neo-commissural alignment during initial TAVI has important clinical implications for future coronary reaccess and aortic valve reintervention, especially in younger patients in whom lifetime treatment of aortic valve and coronary artery disease must be taken into consideration. Although we have shown that a modified delivery system insertion technique during initial valve deployment results in better commissural alignment and less coronary overlap following self-expanding TAVI, further studies are needed to affirm the reproducibility of this strategy. Additionally, unanswered questions remain about the impact of commissural misalignment on balloon-expandable valve-in-valve TAVI, especially in patients with unfavorable aortic root anatomy. It is imperative that clinicians consider these anatomic, device-related, and procedure factors, among others, when evaluating patients for TAVI.

Commissural alignment is particularly important for facilitating coronary reaccess following implantation of the Evolut system since this supra-annular valve extends above the sinotubular junction and coronary ostia. The Evolut valve has a unique “Hat” marker that can be oriented during initial deployment in one of four positions: anterior or posterior in the center of the deployment device (center front or center back) or in the inner/outer curve of the aortic annulus (fluoroscopic insets, bottom left). Note that the “C-tab” is loaded 90^o^ clockwise from the “Hat” marker.

Fluoroscopic images depicting deployment of a 26 mm Evolut Pro valve are shown here. Note that the “Hat” marker is positioned anteriorly in the center of the deployment device (“center front”), with the C-tab seen at the inner curve of the ascending aorta. Multi-detector computed tomography analyses indicate good commissural alignment.
